# Prevalence of Mosquito Populations in the Caribbean Region of Colombia with Important Public Health Implications

**DOI:** 10.3390/tropicalmed8010011

**Published:** 2022-12-25

**Authors:** Eder Cano-Pérez, Martha González-Beltrán, Julia S. Ampuero, Doris Gómez-Camargo, Amy C. Morrison, Helvio Astete

**Affiliations:** 1 Molecular Research Unit (UNIMOL), Faculty of Medicine, University of Cartagena, Cartagena de Indias 130014, Colombia; 2 U.S. Naval Medical Research Unit No. 6, NAMRU-6, Lima 15001, Peru; 3 PhD Program in Tropical Medicine, Faculty of Medicine, University of Cartagena, Cartagena de Indias 130014, Colombia; 4 Department of Pathology, Microbiology, and Immunology, School of Veterinary Medicine, University of California Davis, Davis, CA 95616, USA

**Keywords:** Culicidae, *Aedes*, *Culex*, arbovirus infections, Caribbean region, Colombia

## Abstract

Mosquito studies are important for understanding their role in the transmission of pathogens including arboviruses, parasites, and protozoa. This study characterized the prevalence of Culicidae fauna in rural and peri-urban areas with human populations in the Colombian Caribbean region to establish the risk of transmission of mosquito-borne pathogens. From 2016 to 2017, adult mosquitos were collected in Turbaco (Bolívar), Sabanalarga (Atlántico) and Pueblo Bello (Cesar). The collections in rural areas were in the forest fragments using CDC, Shannon, and human bait traps. In peri-urban areas, Prokopack aspirator collections were used inside households. Entomological and ecological indicators were also calculated. A total of 11,566 mosquito specimens, from 13 genera and 63 species, were collected. The forests fragments of Sabanalarga and Turbaco had the highest species abundance and richness. Turbaco had the highest adult *Aedes aegypti* index. Arbovirus vectors were among the identified species, including *Ae. aegypti*, *Culex quinquefasciatus*, *Haemagogus janthinomys*, *Sabethes chloropterus*, *Aedes angustivittatus*, *Mansonia titillans*, *Coquillettidia venezuelensis* and the subgenera *Culex Melanoconion*. Overall, the diversity and abundance of mosquitoes present in these municipalities establish a potential disease transmission risk by these vectors.

## 1. Introduction

Mosquitoes (Diptera: Culicidae) are one of the most studied groups of arthropods worldwide due to their high transmission capacity for pathogens, including viruses, such as dengue, Zika, and yellow fever, and protozoa, such as *Plasmodium* spp., the causative agents for malaria [1]. Female mosquitoes are hematophagous insects and acquire these pathogens when they feed on infected blood, in turn transmitting them to other healthy hosts [1,2]. According to the World Health Organization (WHO), vector-borne diseases represent more than 17% of all infectious diseases and cause more than 700,000 deaths each year [1,3]. In the Americas, it is estimated that 50% of the population is predisposed to these pathologies [3]; specifically in Colombia, approximately 24% of the inhabitants are at risk of acquiring a disease transmitted by mosquitoes in endemic areas [4]. With the rapid expansion of mosquito distributions due to global warming and increased modes of transportation, epidemiological research of these vectors plays a key role in understanding the populations at risk for contracting diseases transmitted by mosquitos [5,6,7].

The tropical climate of Colombia presents ideal conditions for the migration, adaptation, and persistence of different species of culicids, with approximately 324 species from 28 genera classified throughout the territory [8]. In particular, the Caribbean region contains ecosystems that vary from tropical dry forests to humid mountainous forests with annual rainfall above 3000 mm [9]. This environmental heterogeneity supports the reproduction and colonization of a wide range of mosquitoes in urban, peri-urban, and rural areas. Previous studies in the region have reported the presence of vectors of the genera *Aedes*, *Culex*, *Haemagogus*, *Psorophora*, and *Mansonia* infected with arboviruses in areas deforested for expansion of rice fields and expansion of cattle ranching [10]. In addition, some mosquitoes from forest environments such as species of the genus *Haemagogus* (yellow fever vectors) and *Anopheles* (malaria vectors) have been reported in peri-urban environments in the Caribbean [11]. These studies represent only a fraction of the possible ecological niches in this region of Colombia and have not characterized a wide range of mosquitoes present, especially from the genus *Culex* that are known arbovirus vectors that pose a risk to humans. Hence, the current study aims to characterize the prevalence and biodiversity of *Culex* and other mosquito species over a wider geographic range than previously reported in the Caribbean Region of Colombia, to expand the understanding and geographic range of potential arbovirus vectors in support of arbovirus prevention and control programs in the Region.

## 2. Materials and Methods

### 2.1. Description of the Study Areas

**Figure 1:**
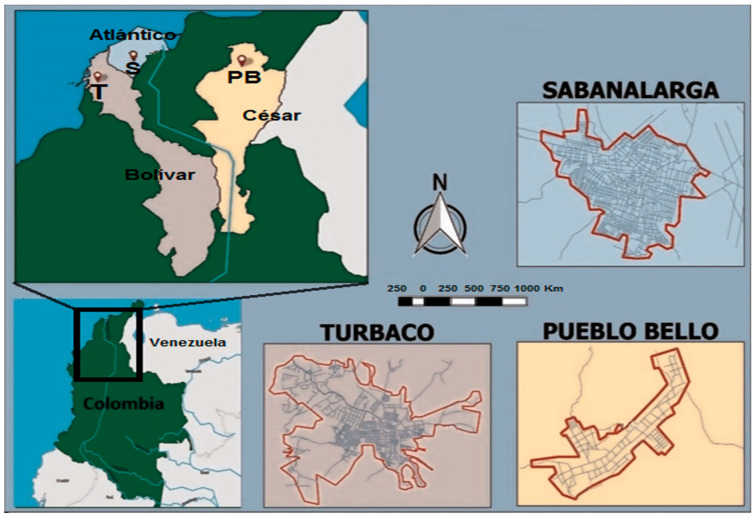
Map of the study sites including the municipalities of Turbaco, Sabanalarga and Pueblo Bello.

The specimen collection areas, corresponding to three municipalities of the Caribbean region of Colombia, are located in the departments of Bolívar (municipality of Turbaco; 10°20′17″ N, 75°24′49″ W), Atlántico (municipality of Sabanalarga; 10°40′48″ N, 74°50′59″ W) and Cesar (municipality of Pueblo Bello; 10°24′38″ N, 73°35′47″ W) (Figure 1). The municipalities of Turbaco and Sabanalarga are municipalities with a dry tropical climate and low or coastal ecotope characterized by large areas of tropical dry forests with altitudes between 90 and 200 m, average temperatures of 27 °C, relative humidity between 80 and 87%, and annual rainfall between 1189 and 1197 mm. In terms of territorial extension, Turbaco has an urban area of 8 km^2^ inhabited by 95,000 people (~11,875 hab/km^2^), while Sabanalarga has an urban area of about 7.5 km^2^ and a population of 67,000 inhabitants (~8933 hab/km^2^). In contrast, Pueblo Bello is a municipality in a high altitude (1200 m) ecotope that is predominantly tropical rainforest, with a temperate climate characterized by an average annual temperature of 21.8 °C, relative humidity of 91.6%, and annual rainfall of 26,572 mm. Pueblo Bello is the municipality with the smallest urban area (about 3.5 km^2^) and population (8236 inhabitants; ~2353 inhabitants/km^2^) among the sampling sites. One of the main characteristics shared by these three municipalities is that they have extensive rural areas with agricultural and livestock exploitation, which are their main economic activities.

### 2.2. Collection and Identification of Specimens

Mosquitoes were collected in four sampling events that occurred over five consecutive days in September 2016, December 2016, May 2017, and September 2017. The samplings covered both rural and peri-urban areas in each municipality.

In this study, rural areas were represented as those areas of forest adjacent to crop fields distant a minimum of 1.5 km from the urban center of each municipality. The collections in these areas occurred between 18:00 and 20:00 using three CDC light traps baited with CO_2_. Each trap was at a maximum distance of 10 m from another. Additionally, human bait and Shannon trap collections with mouth aspirators were also used. Three researchers with personal protection equipment to avoid mosquito bites and minimize risks carried out the human bait collections.

The collections in peri-urban areas were conducted within homes located in peripheral areas of each municipality during daylight hours. These areas were chosen due to the proximity of the houses to wooded areas that facilitate contact with mosquitoes. Sampling of the houses was by convenience and dependent on the acceptance and participation of the inhabitants. After verbal authorization from the homeowners, two investigators collected adult mosquitoes using Prokopack aspirators for 10 min [12]. All the places in the home that the residents allowed access to were inspected (living room, bedrooms, kitchen, bathroom, etc.), when access to the bedrooms was allowed, the aspirator was passed over walls, under furniture, and inside closets and other likely adult mosquito resting sites. Aspiration collections were similarly attempted outside the house from outside walls, under eaves, and outdoor stored materials [13].

All specimens were identified with the available taxonomic keys [14,15,16,17,18,19,20,21] and were preserved in liquid nitrogen until stored at −80 °C at the Tropical Medicine laboratory of the UNIMOL research group of the University of Cartagena. No research permits were required for this study.

### 2.3. Data Analysis

The data were analyzed in two sections. First, the relative abundance of species and ecological indices were calculated from the number of each mosquito species captured in rural areas using PAST v3.26b software. For each sampled fragment, the diversity was estimated using the Shannon–Wiener index, and the species richness was calculated using the Margalef index [22]. The species dominance was assessed using the Simpson index and the equity was estimated using the Pielou index [23,24]. The similarity between the municipalities was calculated using the Jaccard index, which makes comparisons from presence and absence data (qualitative data), and the Bray–Curtis index, which uses abundances (quantitative data) [23]. Second, the frequencies of the different species were calculated from the peri-urban collection sites. The infestation index of *Ae. aegypti* adults (percentage of houses positive for this species) and the density (total of individuals of *Ae. aegypti*/total of houses inspected) were estimated in each locality studied. The chi-square and Kruskal–Wallis tests were applied to compare these variables between municipalities with a statistical significance of 0.05 (*p* < 0.05).

## 3. Results

**Figure 2:**
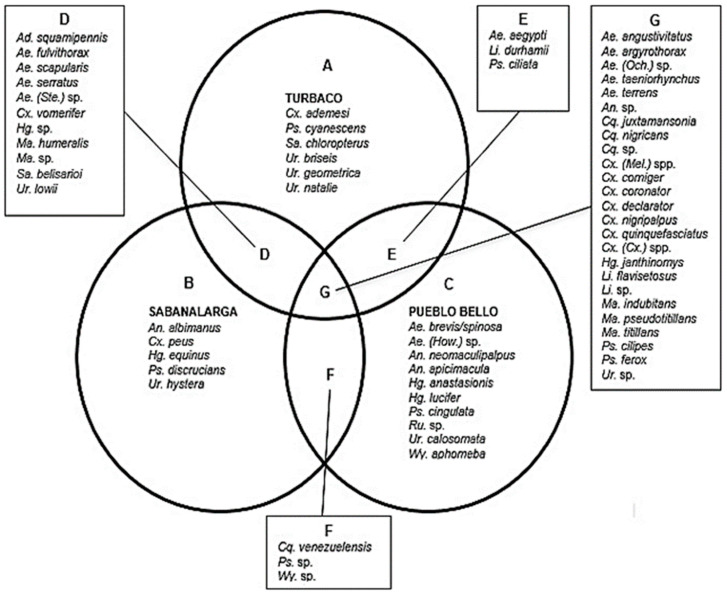
Venn diagram showing the distribution of species found in the different municipalities. (**A**) Turbaco (**B**) Sabanalarga (**C**) Pueblo Bello (**D**) Turbaco–Sabanalarga (**E**) Turbaco–Pueblo Bello (**F**) Sabanalarga–Pueblo Bello (**G**) all municipalities.

In total, 11,566 culicids were captured from Turbaco (*n* = 3935), Sabanalarga (*n* = 4601), and Pueblo Bello (*n* = 3030). A total of two subfamilies, six tribes, 13 genera, and 63 species were identified (Supplementary Table S1, Figure 2). It is important to note that, despite the poorly defined taxonomic status, it was possible to identify at least ten species of the genus *Culex*. Even so, many specimens of the genus *Culex* were only identified up to subgenera (*Culex* and *Melanoconion*); therefore, in this study, they are reported as *Culex (Culex)* spp. and *Culex (Melanoconion)* spp., respectively. Likewise, all specimens that suffered damage or loss of important structures for identification during collection were classified as genus or subgenus.

### 3.1. Collection in Forest Fragments (Rural Area)

The rural samplings in the forest fragments constituted 90.80% of the total material collected (*n* = 10,503), allowing the identification of the 63 species reported in this study. In the municipality of Turbaco, 45 species were found with the most frequent species of *Cx. (Cx)* spp. (27.86%), *Culex quinquefasciatus* (22.26%) and *Aedes scapularis* (12.40%). In the municipality of Sabanalarga, 44 species were collected, finding *Cx. (Mel.)* spp. (24.23%), *Mansonia titillans* (12.45%) and *Coquilletidia nigricans* (11.18%) to be the most frequent species. In Pueblo Bello, 41 species were collected, among which *Aedes angustivittatus* (64.34%) and *Aedes taeniorhynchus* (9.02%) were the most abundant species.

In general, of the 63 species identified, 25 were found in the fragments of all three municipalities; 11 were found only in Turbaco and Sabanalarga; three were identified in Turbaco and Pueblo Bello; and another three species were collected in Sabanalarga and Pueblo Bello. On the other hand, *Culex adamesi, Psorophora cyanescens, Sabethes chloropterus, Uranotaenia briseis, Uranotaenia geometrica* and *Uranotaenia nataliae* were found only in the municipality of Turbaco. Comparatively, the species *Anopheles albimanus*, *Culex peus*, *Haemagogus equinus*, *Psorophora discrucians* and *Uranotaenia hystera* were found exclusively in the municipality of Sabanalarga. On the other hand, ten species were present only in the municipality of Pueblo Bello: *Aedes brevis/spinosa*, *Aedes (Howardina)* sp., *Anopheles neomaculipalpus*, *Anopheles apicimacula*, *Haemagogus anastasionis*, *Haemagogus lucifer*, *Psorophora cingulata*, *Runchomyia* sp., *Uranotaenia calosomata* and *Wyeomyia aphobema* (Figure 2). Among the species mentioned, some correspond to unique collections (only one individual was collected), such as *Ae. (How.)* sp., *Hg. equinus*, *Ps. discrucians*, *Sa. chloropterus*, *Ur. briseis* and *Ur. natalie* (Supplementary Table S1).

The most efficient capture method was human bait, collecting 52.45% (*n* = 5509) of the total captured among the municipalities, followed by the CDC trap with 45.07% (*n* = 4734) and the Shannon trap with 2.47% (*n* = 260). The human bait method found 54 species, followed by the CDC trap with 47, and the Shannon trap with 29. The species abundance and richness for each capture method in each municipality are presented in Supplementary Table S2.

**Figure 3:**
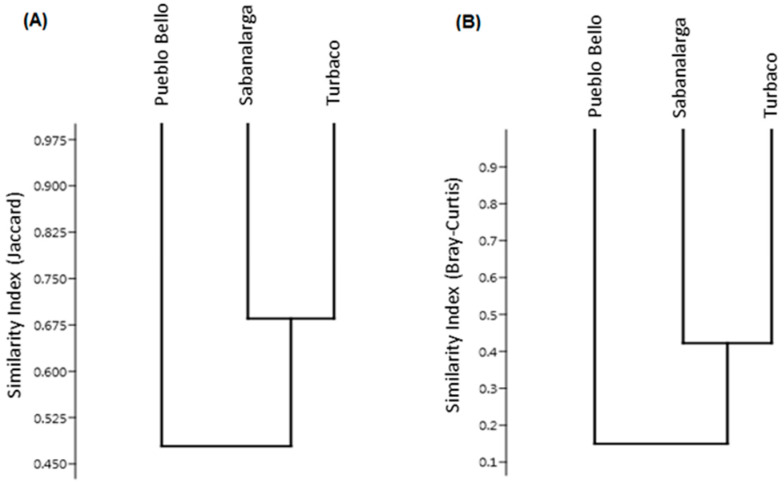
Species similarity dendrograms. (**A**) Jaccard index. (**B**) Bray–Curtis index.

The dendrograms demonstrate that Sabanalarga and Turbaco have a similar composition and abundance of species, with similarity percentages > 40% as determined by the Jaccard and Bray–Curtis indices (Figure 3). These same forest fragments also showed the highest values of diversity and species richness evaluated by the Shannon and Margalef indices. Pueblo Bello had the lowest diversity and richness as calculated by the indices (Table 1). The Simpson index denoted greater dominance in the municipality of Pueblo Bello (0.43), followed by Turbaco (0.15) and Sabanalarga (0.10). Consistently, the Pielou index indicated greater equity in the Sabanalarga fragment (0.70), followed by Turbaco (0.62) and Pueblo Bello (0.39).

### 3.2. Indoor Collections (Peri-urban Area)

In the peri-urban areas, a total of 125 homes were visited in the three municipalities where 1063 total mosquitos from 18 species were collected (Supplementary Table S1). Forty percent of the homes inspected (*n* = 50) among the three municipalities were found to be positive for adult *Ae. aegypti*. There was no statistical difference in the adult infestation index between the three municipalities, although Turbaco was slightly higher than Pueblo Bello and Sabanalarga (chi-square, *p* > 0.05). Likewise, there were no significant differences in the density of adult mosquitoes (Kruskal–Wallis, *p* = 0.567) (Table 2).

In the municipalities of Turbaco and Sabanalarga, the *Ae. aegypti* was the most frequent, totaling 48,24 and 59.3% of the samples corresponding to each municipality. In Pueblo Bello, *Cx. quinquefasciatus* was the most abundant species (59.79%). The indoor samplings also showed the presence of different species of the genera *Culex*, *Aedes*, and *Mansonia* within the houses, as well as the species *Psorophora ciliata, Limatus durhamii,* and *An. neomaculipalpus* (see Supplementary Table S1).

## 4. Discussion

Understanding the presence of culicids in the different regions of Colombia is of great interest to public health. Therefore, this study bridges this gap by enhancing our knowledge of the abundance and diversity of mosquito species present in rural and peri-urban areas in the municipalities of Turbaco (Bolívar), Sabanalarga (Atlántico), and Pueblo Bello (Cesar).

Studies on the mosquito fauna in the Caribbean Region of Colombia are minimal, and thus far, the investigations carried out report a relatively low diversity of species (4–25 identified species) [10,11,25,26]. By contrast, this study collected 63 species of mosquitoes from 13 genera, in this region. The increase in the diversity of species captured may be due to the characteristics of the collection sites, the type of study, the collection techniques, and the taxonomic identification efforts. Unfortunately, the taxonomy of several mosquito species is poorly understood, making it difficult to describe their exact distribution and abundance. This was particularly problematic for the species of the subgenera *Cx (Cx.)* spp. and *Cx (Mel.)* spp. found in this study. Despite extensive taxonomic efforts, it was not possible to identify a high proportion of these mosquitoes. However, the number of *Culex* species that could be identified here (at least ten species) was considerably higher than those reported by the studies mentioned above (zero to two species identified).

In general, the highest abundance and richness of culicids obtained in this study were collected using the human bait technique, followed by the CDC trap and the Shannon trap. Comparatively, these results are similar to those obtained by Parra et al. [27] in a study conducted with mosquitoes from the Urabá, Antioquia. In the former study 59.7% specimens were captured by human bait compared to 52.45% in the current study. However, compared to Parra et al. [27], we captured 18.5% more specimens in CDC traps and 11.2% less in Shannon traps. Additionally, similar results have been obtained in studies conducted on anopheline mosquitoes in Venezuela and Brazil [28,29].

In this study, the forest fragment of the municipality of Turbaco presented the highest species richness, while Sabanalarga had the highest abundance, diversity, and equity (Table 1). This may be due to the presence of more stable and diverse natural breeding sites than those found in other municipalities. Likewise, when determining the similarity between the structure of the mosquito community, Sabanalarga and Turbaco had considerably highest diversity, richness, and equity and a lower dominance index compared to the municipality of Pueblo Bello. These differences are likely because coastal areas with lower altitudes and higher temperatures regularly have a higher abundance and richness of culicids in relation to the more temperate and high zones [30].

It is important to note that the density of *Cx. quinquefasciatus*, a mosquito capable of transmitting multiple diseases, including Venezuelan equine encephalitis and West Nile virus [31,32], was found in the rural environments of all three municipalities. This is of particular concern for public health since this species is usually found in urban environments. These findings are consistent with previous reports from forest areas in Antioquia and Córdoba, indicating *Cx. quinquefasciatus* as a species that effectively adapts to multiple environments [11,27]. Another member of the same genus, *Culex nigripalpus,* was found in these regions. This mosquito, with an ornithophilic tendency, is generally found in dense and humid vegetation, but has also been found to proliferate in urban areas [33]. In the present study, *Cx. nigripalpus* showed a preference for forest spaces in each municipality. However, its proximity to the homes in Turbaco and Pueblo Bello increases the epidemiological importance of this species, which is also involved in the transmission of Venezuelan equine encephalitis and St. Louis encephalitis in Florida [34,35].

The subgenus *Ochlerotatus* of the genus *Aedes* includes some species involved in the transmission of pathogens in humans. This study found the species *Ae. angustivittatus*, a mosquito found in both forest and peri-urban environments. Here, it was found in the rural areas of the three municipalities and in the homes of Turbaco. This species is of importance to public health since it has been reported to be naturally infected by the Venezuelan equine encephalitis virus in Colombia [36]. Additionally, the species *Ae. scapularis* is a culicid found at low elevations and has the ability to reproduce in a wide variety of temporary or semi-temporary fresh waters [18]. Our collections of *Ae. scapularis* were consistent with descriptions as we found this species in the municipalities of Turbaco and Sabanalarga, located at an altitude between 90 and 200 m. This species is also of high importance as a potential vector of various arboviruses, such as Venezuelan equine encephalitis and yellow fever, as well as a secondary vector of bancroftian filariasis [18]. On the other hand, *Ae. taeniorhynchus* is a competent vector for Venezuelan equine encephalitis and the parasite *Dirofilaria immitis* [37,38]. This culicide reproduces in brackish water, so it is considered a species mainly in coastal environments; however, its adaptation to freshwater has led to the colonization of areas far from the sea [39]. This explains the presence of this species in the municipality of Pueblo Bello, which is located approximately 100 km from the coast with an altitude of 1200 m. In addition, its presence in homes in the municipality of Turbaco exemplifies its wide ecological plasticity being found in both urban and rural areas.

Occasional collections of the genus *Haemagogus* were obtained with a mouth aspirator between 18:00 and 18:30 h. Additionally, three specimens were captured in CDC light traps (supplementary Table S2). Species of the genus *Haemagogus* are characterized by diurnal habits and are active at noon hours [19,40]. Although activity and biting have also been reported at approximately 18:00 h [41], this allows us to suspect that the specimens captured by the CDC traps were possibly attracted by CO_2_ in the first minutes after the activation of the traps. This study was directed toward nocturnal and twilight mosquitoes; therefore, no samples were taken during the hours of the day of the greatest activity of the species of this genus, explaining the low density of individuals collected. However, we do report the twilight activity of *Haemagogus* mosquitoes when their habitat is invaded. Among the species found, *Haemagogus janthinomys* and *Hg. equinus* are considered the main vectors of yellow fever in Colombia [25,40]. Here, *Hg. janthinomys* was found in the three municipalities sampled, being more frequent in Turbaco and Sabanalarga. In previous studies, the species *Hg. janthinomys* had been recorded in all the departments that make up the Caribbean region with the exception of the department of Bolívar [10]. However, with these findings, the distribution of this species now extends the entire territory of the region.

Other species with diurnal activity were found, including *Sabethes belisarioi* and *Sa. chloropterus*. The species *Sa. chloropterus* in particular, has been reported as a vector of yellow fever in the Americas [42,43,44]. To complement the studies of diurnal mosquitoes such as those of the genera *Haemagogus* and *Sabethes*, it is necessary to perform vertical sampling between 0 and 30 m, given that the preference of the species of these genera is to the highest strata of the forest [45].

Some other species of health importance identified in our study are *Cq. venezuelensis,* naturally infected with some arboviruses, including Oropouche, Mayaro, and West Nile viruses [46,47]; *Ma. titillans, Mansonia indubitans*, *Ps. cingulata,* and *Psorophora ferox*, considered potential vectors of Venezuelan equine encephalitis [48]. *Ma. titillans* and *Ps. ferox* have also been found to be naturally infected by the St. Louis encephalitis virus [49,50].

The species *Ae. aegypti* was found abundantly in indoor collections due to its known adaptation and preference for urbanized environments [17]. However, it is plausible to consider that the presence of some individuals of *Ae. aegypti* in the forest fragments of the municipalities of Turbaco and Pueblo Bello is due to human activity in the crop fields surrounding the sampled area. *Aedes aegypti* is responsible for the transmission of the urban cycle of arboviruses such as Dengue, Zika, Chikungunya, Yellow fever, and several types of encephalitis [7,51,52,53,54,55,56]. The entomological index established during the home inspections classified Turbaco as the municipality with the highest presence and abundance of *Ae. aegypti* within households. These results can be explained by two observations: first, Turbaco was more urbanized than the other municipalities, which expands the settlement space of the mosquito *Ae. aegypti* in the sampled area. The second reason is attributed to the greater presence of containers with clean stagnant water in the inspected homes of this locality compared to Sabanalarga and Pueblo Bello. Although no immature forms of *Ae. aegypti* were collected in this study, the presence of larvae was observed in these artificial hatcheries.

The municipality of Pueblo Bello was the most rural and was surrounded by forest fragments that presented continuity with the vegetation of the slope. This characteristic generally led to the fact that the dwellings of the inspected neighborhoods were a few meters away from each other and these in turn were surrounded by shrub areas with semi-permanent natural breeding sites such as swamps and puddles. These conditions possibly facilitated the presence of forest species such as *An. neomaculipalpus*, *Li durhamii* and *Ps. ciliata* (Supplementary Table S1). It is important to note that the species *An. neomaculipalpus* is reported in the literature as a potential vector of malaria [57].

The inspected homes were in areas bordering the urban center of each municipality. In addition, some bordered semi-permanent natural breeding sites such as swamps and puddles, suitable for the proliferation of culicid, represent a scenario of easy interaction between these mosquitoes and the surrounding populations. These conditions possibly favored the presence of mosquito species from both peri-urban and rural environments. On the other hand, the 64 species of mosquitoes reported in this study were found in rural areas, some of which are recognized vectors of arboviruses such as dengue, malaria, yellow fever and different types of encephalitis. The rural areas sampled in the three municipalities were generally near areas deforested for the expansion of agriculture and livestock activities, which favors human contact with mosquitos in the area [27]. Therefore, these sampling sites in both peri-urban and rural areas of the Caribbean Region of Colombia undoubtedly pose a potential risk of transmission of diseases that could affect human populations living in or deployed to these regions.

## Figures and Tables

**Table 1:** Ecological indices established in the forest fragments of the municipalities of Turbaco, Sabanalarga and Pueblo Bello.

Municipalities	Ecological Diversity Indices			
	Shannon	Margalef	Pielou	Simpson
Turbaco	2.39	5.40	0.62	0.15
Sabanalarga	2.67	5.11	0.70	0.10
Pueblo Bello	1.48	5.09	0.39	0.43

**Table 2:** Frequency of houses inspected with adult forms of *Ae. aegypti*.

Municipalities	Houses Inspected	Number of Houses with Adult Forms of *Ae. aegypti*	Infestation Index by *Ae. aegypti* (%)	Number of Individuals Collected	Adult Density (Ind/House)
Turbaco	49	21	42.85	246	5.02
Sabanalarga	32	12	37.50	51	1.59
Pueblo Bello	44	17	38.63	105	2.39
Total	125	50	40.00	402	3.21

## Data Availability

All data used to support our findings are included in the manuscript. The corresponding author may provide any additional request information.

## Supplementary Materials

The following supporting information can be downloaded at: https://www.mdpi.com/article/10.3390/tropicalmed8010011/s1, Table S1: Distribution and frequency of mosquito species in rural and urban areas from three municipalities; Table S2: Distribution of species according to collection method in rural areas.
