# Supplementary material for: Prevalence of Mosquito Populations in the Caribbean Region of Colombia with Important Public Health Implications
Source: Trop Med Infect Dis. 2022 Dec 25;8(1):11. doi: 10.3390/tropicalmed8010011 (PMC9867490; doi:10.3390/tropicalmed8010011)
Supplement: Supplementary file 1 [file tropicalmed-08-00011-s001.zip › tropicalmed-2101954-supplementary.pdf]

Supplementary material

Prevalence of mosquito populations in the Caribbean Region of Colombia with important Public Health implications

Eder Cano-Pérez 1,*, Martha González-Beltrán 1, Julia S. Ampuero 2, Doris Gómez-Camargo 1,3, Amy C. Morrison 4, Helvio Astete 2

Supplementary table S1. Distribution and frequency of mosquito species in rural and urban areas from three municipalities.

Species	Rural area							Periurban area							Total	%
	Turbaco		Sabanalarga		Pueblo Bello		Sub total	Turbaco		Sabanalarga		Pueblo Bello		Sub total		
	N	%	N	%	N	%		N	%	N	%	N	%			
Aedeomyia (Aedeomyia) squamipennis	20	0,58	47	1,04	0	0,00	67								67	0,58
Aedes (Howardina) brevis/spinosa	0	0,00	0	0,00	3	0,12	3								3	0,03
Aedes (How.) fulvithorax	25	0,73	7	0,16	0	0,00	32								32	0,28
Aedes (How.) sp.	0	0,00	0	0,00	1	0,04	1								1	0,01
Aedes (Ochlerotatus) angustivittatus	6	0,18	72	1,59	1647	64,34	1725	3	0,59	0	0,00	0	0,00	3	1728	14,94
Aedes (Och.) argyrothorax	3	0,09	39	0,86	1	0,04	43	1	0,20	0	0,00	1	0,21	2	45	0,39
Aedes (Och.) scapularis	425	12,40	171	3,79	0	0,00	596								596	5,15
Aedes (Och.) serratus	16	0,47	10	0,22	0	0,00	26								26	0,22
Aedes (Och.) sp.	19	0,55	63	1,40	182	7,11	264	0	0,00	0	0,00	1	0,21	1	265	2,29
Aedes (Och.) taeniorhynchus	27	0,79	44	0,97	231	9,02	302	1	0,20	0	0,00	0	0,00	1	303	2,62
Aedes (Protomacleaya) terrens	2	0,06	22	0,49	10	0,39	34	0	0,00	0	0,00	2	0,43	2	36	0,31
Aedes (Stegomyia) aegypti	3	0,09	0	0,00	1	0,04	4	246	48,52	51	59,30	105	22,34	402	406	3,51
Aedes (Ste.) sp.	5	0,15	3	0,07	0	0,00	8	2	0,39	2	2,33	0	0,00	4	12	0,10

Anopheles (Anopheles) apicimacula	0	0,00	0	0,00	5	0,20	5								5	0,04
Anopheles (Ano.) neomaculipalpus	0	0,00	0	0,00	8	0,31	8	0	0,00	0	0,00	2	0,43	2	10	0,09
Anopheles (Ano.) sp.	1	0,03	9	0,20	22	0,86	32								32	0,28
Anopheles (Nyssorhynchus) albimanus	0	0,00	2	0,04	0	0,00	2								2	0,02
Coquillettidia (Rhynchotaenia) juxtamansonia	6	0,18	280	6,20	7	0,27	293								293	2,53
Coquillettidia (Rhy.) nigricans	71	2,07	505	11,18	1	0,04	577								577	4,99
Coquillettidia (Rhy.) sp.	7	0,20	289	6,40	6	0,23	302								302	2,61
Coquillettidia (Rhy.) venezuelensis	0	0,00	31	0,69	43	1,68	74								74	0,64
Culex (Culex) coronator	99	2,89	118	2,61	33	1,29	250	7	1,38	3	3,49	5	1,06	15	265	2,29
Culex (Cx.) declarator	83	2,42	75	1,66	11	0,43	169	22	4,34	2	2,33	6	1,28	30	199	1,72
Culex (Cx.) nigripalpus	125	3,65	108	2,39	31	1,21	264	2	0,39	0	0	4	0,85	6	270	2,33
Culex (Cx.) peus	0	0,00	4	0,09	0	0,00	4								4	0,03
Culex (Cx.) quinquefasciatus	763	22,26	239	5,29	208	8,13	1210	134	26,43	18	20,93	281	59,79	433	1643	14,21
Culex (Cx.) spp.	955	27,86	294	6,51	25	0,98	1274	66	13,02	4	4,65	61	12,98	131	1405	12,15
Culex (Melanoconion) adamesi	2	0,06	0	0,00	0	0,00	2								2	0,02
Culex (Mel.) spp.	158	4,61	1094	24,23	1	0,04	1253								1253	10,83
Culex (Mel.) vomerifer	3	0,09	3	0,07	0	0,00	6								6	0,05
Culex (Phenacomyia) corniger	37	1,08	5	0,11	5	0,20	47	23	4,54	0	0,00	0	0,00	23	70	0,61
Haemagogus (Haemagogus) anastasionis	0	0,00	0	0,00	7	0,27	7								7	0,06
Haemagogus (Hae.) equinus	0	0,00	1	0,02	0	0,00	1								1	0,01
Haemagogus (Hae.) janthinomys	22	0,64	20	0,44	1	0,04	43								43	0,37
Haemagogus (Hae.) lucifer	0	0,00	0	0,00	6	0,23	6								6	0,05
Haemagogus (Hae.) sp.	1	0,03	2	0,04	0	0,00	3								3	0,03

Limatus durhamii	1	0,03	0	0,00	4	0,16	5	0	0,00	0	0,0	1	0,21	1	6	0,05
Limatus flavisetosus	3	0,09	1	0,02	1	0,04	5								5	0,04
Limatus sp.	1	0,03	2	0,04	2	0,08	5								5	0,04
Mansonia (Man.) humeralis	41	1,20	76	1,68	0	0,00	117	0	0,00	1	1,16	0	0,00	1	118	1,02
Mansonia (Man.) indubitans	120	3,50	165	3,65	1	0,04	286								286	2,47
Mansonia (Man.) pseudotitillans	10	0,29	87	1,93	2	0,08	99								99	0,86
Mansonia (Man.) sp.	8	0,23	7	0,16	0	0,00	15								15	0,13
Mansonia (Man.) titillans	124	3,62	562	12,45	1	0,04	687	0	0,00	5	5,81	0,0	0,00	5	692	5,98
Psorophora (Grabhamia) cingulata	0	0,00	0	0,00	8	0,31	8								8	0,07
Psorophora (Janthinosoma) cyanescens	153	4,46	0	0,00	0	0,00	153								153	1,32
Psorophora (Jan.) discrucians	0	0,00	1	0,02	0	0,00	1								1	0,01
Psorophora (Jan.) ferox	14	0,41	21	0,47	4	0,16	39								39	0,34
Psorophora (Jan.) sp.	0	0,00	13	0,29	12	0,47	25								25	0,22
Psorophora (Psorophora) ciliata	1	0,03	0	0,00	3	0,12	4	0	0,00	0	0,0	1	0,21	1	5	0,04
Psorophora (Pso.) cilipes	1	0,03	1	0,02	2	0,08	4								4	0,03
Runchomyia sp.	0	0,00	0	0,00	8	0,31	8								8	0,07
Sabethes (Sabethes) belisarioi	3	0,09	4	0,09	0	0,00	7								7	0,06
Sabethes (Sabethoides) chloropterus	1	0,03	0	0,00	0	0,00	1								1	0,01
Uranotaenia (Uranotaenia) briseis	1	0,03	0	0,00	0	0,00	1								1	0,01
Uranotaenia (Ura.) calosomata	0	0,00	0	0,00	3	0,12	3								3	0,03
Uranotaenia (Ura.) geometrica	50	1,46	0	0,00	0	0,00	50								50	0,43
Uranotaenia (Ura.) lowii	2	0,06	12	0,27	0	0,00	14								14	0,12

Uranotaenia (Ura.) nataliae	1	0,03	0	0,00	0	0,00	1								1	0,01
Uranotaenia (Ura.) sp.	9	0,26	2	0,04	1	0,04	12								12	0,10
Uranotenia (Ura.) hystera	0	0,00	3	0,07	0	0,00	3								3	0,03
Wyeomyia (Dodecamyia) aphobema	0	0,00	0	0,00	2	0,08	2								2	0,02
Wyeomyia sp.	0	0,00	1	0,02	10	0,39	11								11	0,10
**Total number of individuals**	**3428**	**100**	**4515**	**100**	**2560**	**100**	**10503**	**507**	**100**	**86**	**100**	**470**	**100**	**1063**	**11566**	**100**
**Total number of species**	**45**		**44**		**41**		**63**	**11**		**8**		**12**			**18**	

Supplementary table S2. Distribution of species according to collection method in rural areas.

Species	Turbaco			Total	Sabanalarga			Total	Pueblo Bello			Total
	CDC	HB*	ST†		CDC	HB	ST		CDC	HB	ST	
Ad. (Aed.) squamipennis	1	15	4	20	47	0	0	47				
Ae. (How) brevis/spinosa									0	3	0	3
Ae. (How.) fulvithorax	3	22	0	25	0	7	0	7				
Ae. (How) sp.									0	1	0	1
Ae. (Och.) angustivittatus	2	4	0	6	9	59	4	72	109	1538	0	1647
Ae. (Och.) argyrothorax	1	2	0	3	8	31	0	39	0	1	0	1
Ae. (Och.) scapularis	79	346	0	425	57	112	2	171				
Ae. (Och.) serratus	16	0	0	16	0	10	0	10				
Ae. (Och.) sp.	14	4	1	19	34	28	1	63	2	180	0	182
Ae. (Och.) taeniorhynchus	12	12	3	27	10	13	21	44	25	204	2	231
Ae. (Pro.) terrens	2	0	0	2	12	10	0	22	3	7	0	10
Ae. (Ste.) aegypti	1	2	0	3					0	1	0	1
Ae. (Ste.) sp.	5	0	0	5	2	1	0	3				
An. (Ano.) apicimacula									0	5	0	5
An. (Ano.) neomaculipalpus									4	0	4	8
An. (Ano.) sp.	1	0	0	1	5	4	0	9	1	21	0	22
An. (Nys.) albimanus					1	1	0	2				
Cq. (Rhy.) Juxtamansonia	3	2	1	6	102	178	0	280	1	6	0	7
Cq. (Rhy.) nigricans	31	40	0	71	159	346	0	505	1	0	0	1
Cq. (Rhy.) sp.	3	3	1	7	139	140	10	289	0	6	0	6
Cq. (Rhy.) venezuelensis					8	23	0	31	3	38	2	43
Cx. (Cx.) coronator	54	45	0	99	49	60	9	118	10	20	3	33
Cx. (Cx.) declarator	52	23	8	83	40	19	16	75		10	1	11
Cx. (Cx.) nigripalpus	98	25	2	125	37	68	3	108	5	26	0	31
Cx. (Cx.) peus					4	0	0	4				
Cx. (Cx.) quinquefasciatus	688	57	18	763	146	75	18	239	39	160	9	208
Cx. (Cx) spp.	754	198	3	955	238	50	6	294	14	11	0	25
Cx. (Mel.) adamesi	2	0	0	2								
Cx. (Mel.) spp.	115	40	3	158	770	320	4	1094	0	1	0	1
Cx. (Mel.) vomerifer	3	0	0	3	3	0	0	3				
Cx. (Phe.) corniger	29	7	1	37	3	0	2	5	1	2	2	5
Hg. (Hae.) anastasionis									0	7	0	7
Hg. (Hae.) equinus					0	1	0	1				
Hg. (Hae.) janthinomys	2	20	0	22	1	17	2	20	0	1	0	1
Hg. (Hae.) lucifer									0	6	0	6
Hg. (Hae.) sp.	0	1	0	1	0	2	0	2				
Li. durhamii	0	1	0	1					0	4	0	4
Li. flavisetosus	3	0	0	3	1	0	0	1	0	1	0	1
Li. sp.			1	1	2	0	0	2	0	2	0	2
Ma. (Man) humeralis	36	3	2	41	76	0	0	76				
Ma. (Man) indubitans	39	79	2	120	91	74	0	165	0	1	0	1

Ma. (Man) pseudotitillans	4	6	0	10	43	44	0	87	0	2	0	2
Ma. (Man) sp.	5	3	0	8	2	3	2	7				
Ma. (Man) titillans	72	50	2	124	230	314	18	562	0	1	0	1
Ps. (Gra.) cingulata									0	1	7	8
Ps. (Jan.) cyanescens	17	135	1	153								
Ps. (Jan.) discrucians					0	1	0	1				
Ps. (Jan.) ferox	1	7	6	14	3	16	2	21	0	4	0	4
Ps. (Jan.) sp.					11	1	1	13	0	12	0	12
Ps. (Pso.) ciliata	0	1	0	1					0	3	0	3
Ps. (Pso.) cilipes	1	0	0	1	0	1	0	1	0	2	0	2
Ru. sp.									0	8	0	8
Sa. (Sab.) belisarioi	0	3	0	3	0	4	0	4				
Sa. (Sab.) chloropterus	0	1	0	1								
Ur. (Ura.) briseis	1	0	0	1								
Ur. (Ura.) calosomata									3	0	0	3
Ur. (Ura.) geometrica	1	1	48	50								
Ur. (Ura.) lowii	2	0	0	2	3	9	0	12				
Ur. (Ura.) nataliae	1	0	0	1								
Ur. (Ura.) sp.	9	0	0	9	0	1	1	2	1	0	0	1
Ur. (Ura.) hystera					3	0	0	3				
Wy. (Dod.) aphobema									0	2	0	2
Wy. sp.						1		1	0	9	1	10
**Total number of individuals**	**2163**	**1158**	**107**	**3428**	**2349**	**2044**	**122**	**4515**	**222**	**2307**	**31**	**2560**
**Total number of species**	**39**	**32**	**18**	**45**	**35**	**36**	**18**	**44**	**16**	**37**	**9**	**41**

*HB: human bait
†ST: Shannon trap
